# Functional outcomes of extra-articular scapula fracture fixation with distal humeral Y-type locking plate: a retrospective study

**DOI:** 10.1186/s13018-019-1205-y

**Published:** 2019-06-13

**Authors:** Yuanjun Hu, Huiming Shi, Fei Wang, Guangtie Ren, Ruiping Cheng, Zhizhong Zhang

**Affiliations:** Traumatic Orthopaedics Department Ward I, Hanzhong Central Hospital, Hanzhong, 723000 Shaanxi China

**Keywords:** Scapular fracture, Open reduction, Y-type locking plate, Reconstruction locking plate

## Abstract

**Background:**

This study aimed to compare the functional outcomes of the Y-type locking plate with the straight reconstruction locking plate for severe displaced scapular fractures.

**Methods:**

This was a retrospective cohort study of 37 patients with severe displaced scapular body and neck fractures treated between July 2013 and October 2016 at the Hanzhong Central Hospital. Treatment selection was based on the surgeon’s experience and discussion with the patient. Sixteen patients received Y-type locking plates and 21 patients received straight reconstruction locking plates. The primary indexes were the Constant Shoulder Function (CSF) and Disabilities of the arm, shoulder, and hand (DASH) scores at 3, 6, and 12 months.

**Results:**

There were 32 males and five females. Mean age was 46.0 ± 10.0 years. The cause of injury included car accident, fall, high fall, and bruising. At 3 months, compared with the straight reconstruction locking plate group, the Y-type locking plate group showed higher CSF scores (82.9 ± 3.5 vs. 79.3 ± 4.4, *P* = 0.01) and lower DASH scores (9.5 ± 2.5 vs. 12.7 ± 3.9, *P* = 0.008). There were no differences at 6 and 12 months. There were no differences between the two groups regarding intraoperative blood loss (*P* = 0.65) and operation time (*P* = 0.634). There were no complications such as plate rupture and screw prolapse during the 1-year follow-up.

**Conclusions:**

Open reduction using the distal humeral Y-type locking plate can achieve better short-term functional outcomes (3 months) than the straight reconstruction locking plate for severe displaced scapular body and neck fractures, but outcomes are similar at 6 and 12 months. Level of evidence: II-2.

## Background

Scapular fractures are relatively uncommon, accounting for 0.4–1% of all fractures and 3–5% of upper extremity fractures [1–5]. Scapular fractures usually result from high-energy trauma [6]. Body and neck fractures account for 62% to 98% of all scapular fractures, respectively [5, 7, 8]. Most of the scapular fractures are treated with conservative treatment [6, 9–11], but conservative treatment for severe displaced scapular fractures can lead to shoulder joint dysfunction, chronic pain, and other complications [12, 13]. For extra-articular scapular fractures, the rate of poor functional outcome after conservative treatment is 20%, the rate of radiographic scapula deformity is 25%, and pain is present in 12% of the patients [10].

The literature about the internal fixation of scapular fractures presents a wide variety of approaches [3–5, 7, 13–16], but the current approaches with locking plates are challenging because of the shape of the scapula, resulting in suboptimal patient outcomes. Clinical observations have shown that most unstable fractures of the scapula are comminuted extra-articular fractures of the scapular body or glenoid neck. No plate is specifically available for the fixation of such complex fractures. The most commonly used plates include locking compression plate, reconstruction plate, T-shape plate, calcaneous deformed plate, and microplate [3–5, 7, 13, 15].

In the present study, a distal humeral Y-type locking plate (Y-type locking compression plate 39°, AZX-LL, with 3.5-mm locking nails) was chosen for the fixation of fractures of the scapular body and glenoid neck. Indeed, the scapula is thinning below the glenoid neck, but is thickening at the external margin. After remodeling, the proximal part of the Y-type locking plate is V-shaped, of which one side could be closely fixed to the glenoid neck, and the other side could be fixed to the basis of the scapular spine. The distal part of the Y-type locking plate could be fixed to the external margin of the scapula, and therefore provides triangular multi-plane support for the fixation. Such fixation could turn the unstable fracture into a whole. Thus the fixation could be relatively stable and secure, which could provide material basis for early functional exercises after surgery.

A Y-type locking plate could overcome some of the challenges encountered with the traditional straight reconstruction locking plate, but there is no literature on the Y-type locking plate for the treatment of scapular fractures. Therefore, this study compared the functional outcomes of the Y-type locking plate with the straight reconstruction locking plate for the treatment of severe displaced scapular fractures. The results should provide a theoretical basis for clinical orthopedists for the selection of the most appropriate type of plate for this type of fractures.

## Methods

### Study design and patients

This was a retrospective cohort study of 37 patients with severe displaced scapular body and neck fractures treated between July 2013 and October 2016 at the Orthopedics Department of Hanzhong Central Hospital. The study was approved by the ethics committee of the Hanzhong Central Hospital (#IRB2018-S; April 12th, 2018). The need for individual consent was waived by the committee.

The indication for surgery were (i) medial/lateral displacement ≥ 20 mm, (ii) angular deformity ≥ 45°, (iii) glenopolar angle (GPA) ≤ 22°, (iv) medial/lateral displacement ≥ 15 mm and angulation ≥ 30°, and (v) double disruption of the superior shoulder suspensory complex with displacement ≥ 10 mm [6–8, 17]. At the time of surgery, the selection of treatment was based on the experience of the surgeon and after discussion with the patient. Sixteen patients received the distal humeral Y-type locking plate and 21 patients received a straight reconstruction locking plate (control group). All patients were treated at the Hanzhong Central Hospital and were identified using the central electronic patient database. The database was searched in January 2017 for patients with scapular fracture and the patients were screened using the eligibility criteria.

The inclusion criteria were (1) extra-articular fracture of the scapular body or glenoid neck according to Cole’s criteria [6], i.e., fractures not involving the articular processes of the scapula, with AO/OTA type 14-A3.1 or 3.2 [18, 19]; (2) 18–70 years of age; (3) ≤ 21 days between injury and surgery [6, 17]; (4) indications for surgical reduction (as above); and (5) available follow-up at 3, 6, and 12 months. The exclusion criteria were (1) intra-articular fractures, (2) preoperative nerve injury, (3) patients who could not perform functional exercises after surgery, or (4) patients with missing data.

### Data collection

After identification of the patients, the medical charts were reviewed for confirmation of the inclusion and exclusion criteria. Demographics, injury characteristics (location, cause, combined injuries, and number of days between injury and surgery), surgical characteristics (blood loss and operation time), and follow-up data (Constant Shoulder Function (CSF) score and the Disabilities of the arm, shoulder, and hand (DASH)) were extracted from the medical charts by two authors independently. Data extraction was compared and discrepancies were discussed.

### Evaluation of the scapular fracture and 3D modeling

**Fig. 1:**
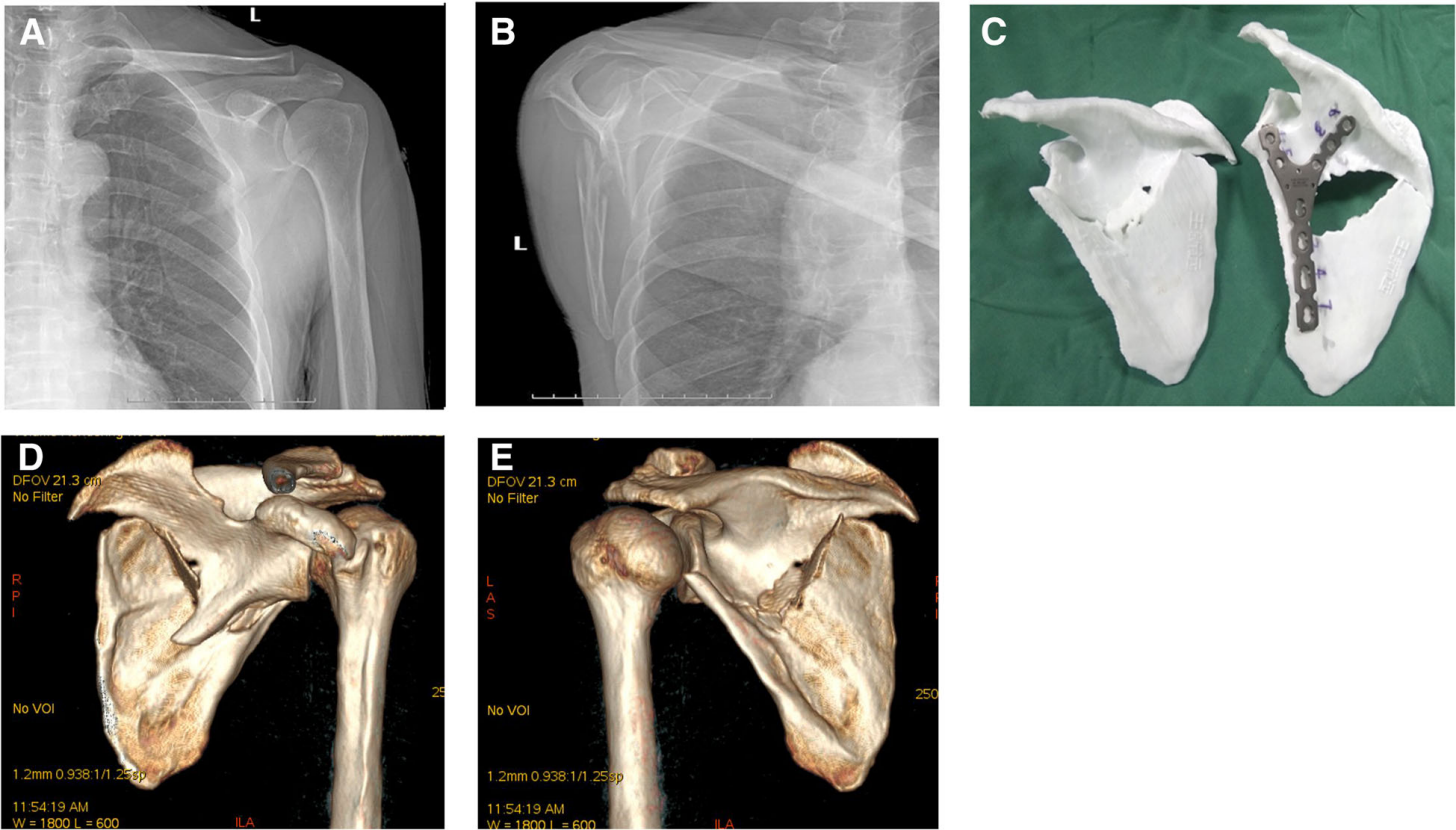
A 61-year-old man sustained a left scapular body and glenoid neck fracture (AO 14-A3.1) associated with multiple left rib fractures. **a** GPA was 26.4° on the anteroposterior X-ray film of the scapula fracture. **b** The lateral displacement was 33.2 mm (defined as displacement between the proximal and distal fragments at the lateral border) and angular deformity was 34.3° on the lateral X-ray film. **c** 3D model, fracture reduction, and preoperative repair planning. **d**–**e** Three-dimensional computed tomography reconstruction of the scapula fracture

The preoperative radiographic evaluation included anteroposterior (Fig. 1a) and lateral (Fig. 1b) X-rays. Computed tomography (SOMATOM Definition Flash, scan thickness 1.0 mm, 128 rows) scans with three-dimensional reconstructions (3D-CT scans) were ordered when a displaced scapular fracture was diagnosed. The 3D-CT data were entered in the 3D printing software studio (Xijin Zhenwo 3D printing cloud terminal, reconstruction software: Mimics 17.0). The 3D fracture model of the scapula with the same size and the mirror model of the contralateral scapula were printed for all patients. The reduction and fixation strategy were designed according to the 3D fracture and mirror model. The Y-type locking plate (Fig. 1c) or straight reconstruction locking plate was pre-bent according to the 3D mirror model. 3D printing was carried out using polylactic acid (PLA) (Weinan Dingxin Chuangxin Zhizao Technology Co., Ltd., Beijing, China).

The Y-type locking plate was from Beijing Best Biotech Co., Ltd. (Beijing, China): 39°, model number AZX-LL, thickness of 2.4 mm, and 3.5-mm locking nails. The straight reconstruction locking plate was from Beijing Besida Biotechnology Co., Ltd. (Beijing, China): model AZX-LL, thickness of 3 mm, and 3.5-mm locking nails. The straight reconstruction locking plate costs 5530 Yuan and the Y-type locking plate costs 5600 Yuan. The price of the two plates is comparable and did not significantly affect the selection of the treatment by the patients.

### Surgical technique

**Fig. 2:**
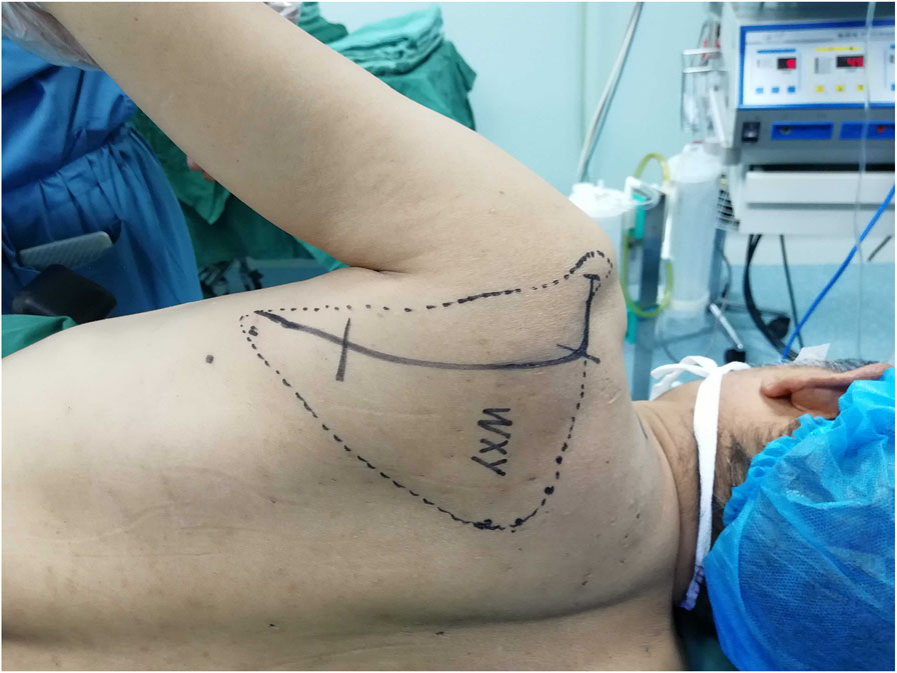
The modified Judet approach

**Fig. 3:**
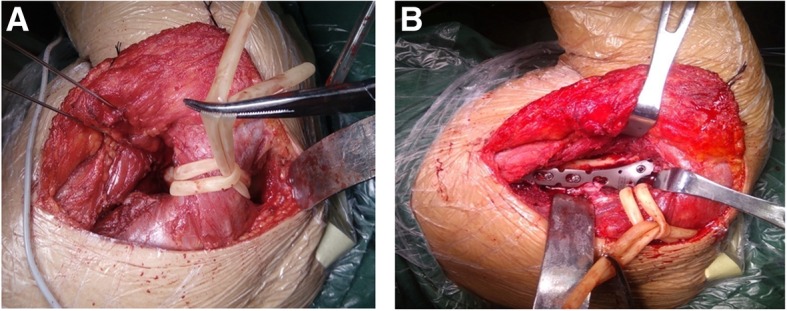
**a** The tunnel was established using a rubber strip pulling between the deltoid muscle and the infraspinatus muscle. **b** The plate was implanted through the inferior muscle channel

All patients were treated by the two same surgeons, both with at least 20 years of experience (H.S., deputy chief physician; and Z.Z., deputy chief physician). The procedure was performed under general anesthesia. The patients were placed in the lateral decubitus position on the healthy side. The affected shoulder was disinfected and the upper limb was wrapped in aseptic drape. The modified Judet approach was used to expose the scapular fracture [15, 20] (Fig. 2). An L-shaped incision was made from the distal tip of the acromion, parallel to the scapular spine, along the lateral border of the scapula, to the inferior scapular angle. The skin, subcutaneous tissue, and fascia were incised to expose the posterior part of the deltoid muscle. The interval between the teres minor and infraspinatus showed the lateral border of the scapula. The circumflex scapular artery was embedded between the two ends of the scapular body fracture, so the artery was ligated to expose the parts of the scapular body and glenoid neck fracture. The glenoid neck of the scapula is covered by the infraspinatus and the deltoid muscle. The two muscles were bluntly dissected at the lateral origin of the scapular spine, opening a window to form submuscle tunnels. Rubber strips were used to pull the posterior part of the deltoid muscle and infraspinatus muscle (Fig. 3a), while protecting the neurovascular bundle, therefore revealing the base of the scapular spine and the scapular glenoid neck. Referring to the 3D model and according to the preoperative planning, the fracture after reduction was temporarily fixed using Kirschner wires. For the Y-type group, a Y-type locking plate was implanted along the lateral border of the scapula via the submuscle tunnels (Fig. 3b). For the straight reconstruction locking plate group, the straight reconstruction locking plate was placed along the lateral border of the scapula. The shoulder joint was repeatedly moved and intraoperative C-arm fluoroscopy was performed to see if the screws penetrated into the joint cavity. Drainage was placed and the incision was closed in a layered fashion.

### Postoperative follow-up

In the Y-type locking plate group, the limb was immobilized for 2 weeks, with functional exercises of the elbow, wrist, and fingers in the meantime. Passive shoulder-lift and pendulum-like exercises were performed after 1 week. After 2 weeks, the patients progressively exercised with active forward flexion, extension, and abduction of the shoulder joint. After 3 weeks, the shoulder joints were gradually lifted. After 6 weeks, shoulder muscle strength training and endurance training were gradually performed.

In the straight reconstruction locking plate group, the limbs were suspended for 4 weeks, with functional exercises of the elbow, wrist, and fingers. Passive shoulder-lift and pendulum-like exercises were performed after 2 week. After 4 weeks, the patients progressively exercised with active forward flexion, posterior extension, and abduction of the shoulder joint, gradually increasing the range of joint motion. After 6 weeks, shoulder muscle strength training and endurance training were gradually performed.

**Fig. 4:**
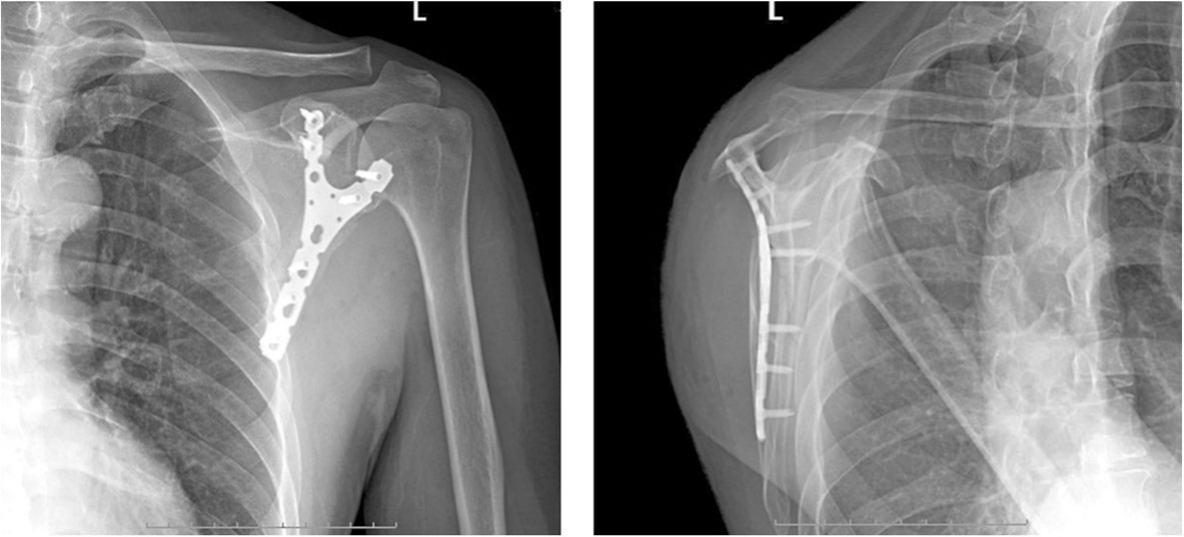
Postoperative scapular X-ray radiographies of the implanted Y-type locking plate in the anteroposterior and lateral positions

**Fig. 5:**
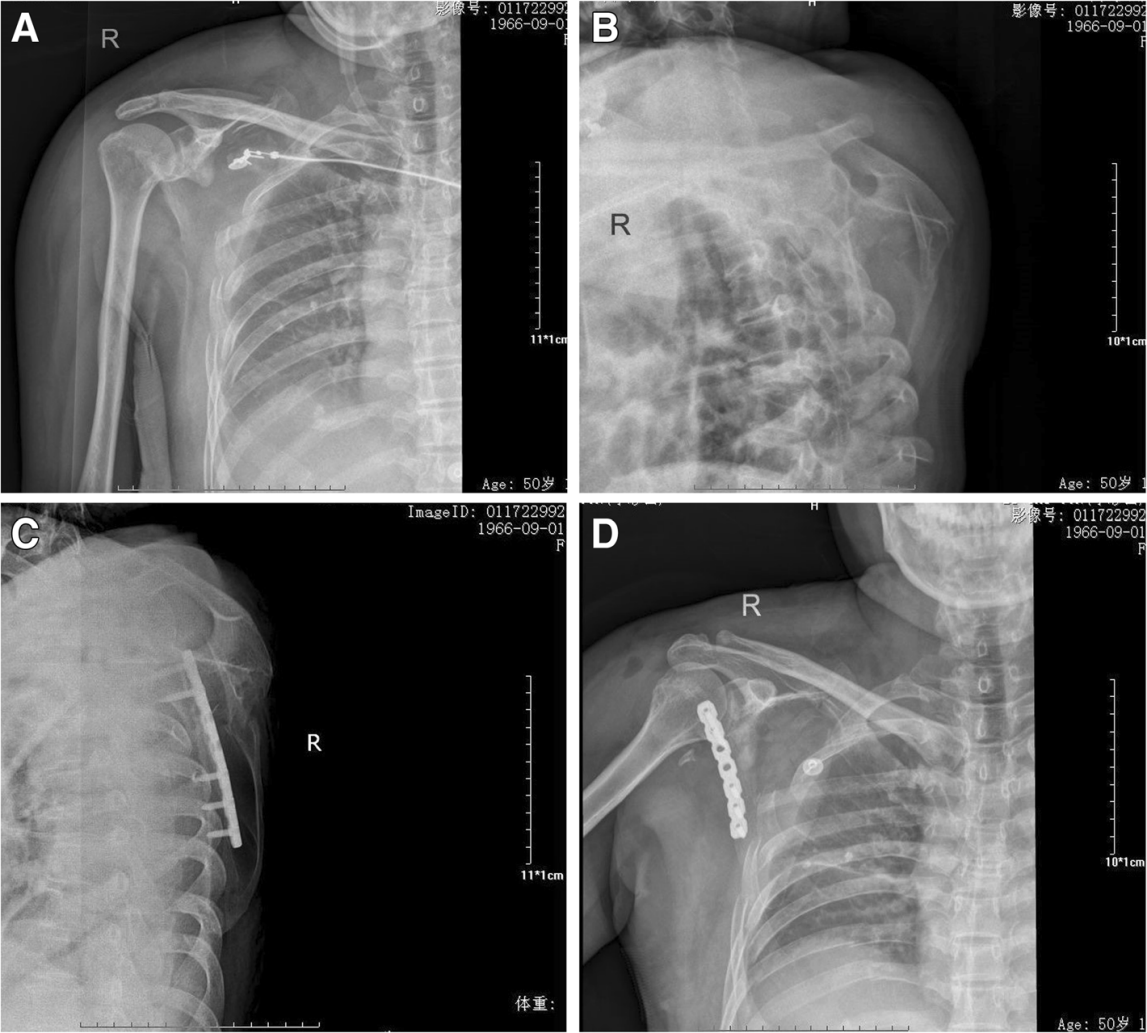
A 50-year-old female patient with fractures of the right scapula (AO 14A-3.1) accompanied by multiple fractured ribs caused by car accident. The GPA on the posteroanterior picture showed that the angle was 28.8° and facture displacement was 3.5 cm. Straight reconstruction locking plate was used for the fixation. **a** Posteroanterior X-ray before surgery. **b** Lateral X-ray before surgery. **c** Posteroanterior X-ray after surgery. **d** Lateral X-ray after surgery

All patients were followed at 1, 3, 6, and 12 months after surgery using X-ray with anteroposterior and lateral view (Figs. 4 and 5). The CSF score and the DASH questionnaire were evaluated at 3, 6, and 12 months [14, 21].

### Outcomes

The exposure variable was the type of plate, Y-type locking plate vs. straight reconstruction locking plate. The primary outcomes were the CSF and DASH scores at 3, 6, and 12 months [14, 21]. The secondary outcomes were intraoperative blood loss, operation time, postoperative complications (such as wound healing, infection, nonunion, implant failure, pain, and plate prominence), and demographic and clinical data (gender, age, location of injury, cause of injury, combined injury, and preoperative days) were compared between the two groups.

### Sample size

This was a retrospective study of all patients treated between July 2013 and October 2016 at the Orthopedics Department of Hanzhong Central Hospital and matching the inclusion and exclusion criteria.

### Statistical analysis

Grouping was based on the type of locking plate used for the repair of the scapular fracture. The distribution of the continuous data was assessed using the Kolmogorov-Smirnov test. Normally distributed data are presented as means ± standard deviation and were analyzed using the Student *t* test. Non-normally distributed data are presented as median (range) and were analyzed using the Mann-Whitney U test. Categorical variables are presented as frequencies and were analyzed using the Fisher’s exact test. SPSS 17.0 (IBM, Armonk, NY, USA) was used for all analyses. Two-sided*P* values < 0.05 were considered to be statistically significant. Patients with missing data or missing follow-up at 3, 6, or 12 months were excluded. No sensitivity analysis could be performed.

## Results

### Characteristics of the patients

Thirty-seven patients were included as per the inclusion/exclusion criteria. All patients were Chinese. The 37 patients included 32 males and five females. Mean age was 46.0 ± 10.0 (range, 26–64) years. The fractures were on the left side in 22 patients and on the right side in 15. The cause of injury included car accident in 19 patients, fall in eight, high fall in six, and bruising in four. The combined injuries included combined thoracic injuries with multiple rib fractures in 24 patients; combined craniocerebral injuries in six; combined with ipsilateral clavicle fracture in six; combined with abdominal injuries in three; and combined with fractures in other sites in two. There were six patients without combined injuries.

There were no significant differences in gender, age, location of injury, cause of injury, combined injury, and preoperative days between the two groups (all *P* > 0.05) (Table 1). All patients were followed up for at least 12 months.

**Table 1:** Characteristics of the 37 patients with extra-articular scapular fracture

	Y-type locking plate *n* = 16	Straight reconstruction locking plate *n* = 21	*P*
Age (years), mean ± SD	45.6 ± 9.3	46.3 ± 10.7	0.831
Gender, *n* (%)			
Male	14 (87)	18 (86)	0.875
Female	2 (13)	3 (14)	
Preoperative days, mean ± SD	8.1 ± 3.7	7.3 ± 3.9	0.542
Location of injury, *n* (%)			
Left	7 (44)	15 (71)	0.089
Right	9 (66)	6 (29)	
Cause of injury, *n* (%)			
Traffic accident injuries	8 (50)	11 (52)	0.959
Fall injuries	3 (19)	5 (24)	
High fall	3 (19)	3 (14)	
Bruises	2 (13)	2 (10)	
Combined injuries, *n* (%)^a^			
a	6 (38)	8 (38)	0.289
b	0	3 (14)	
a + b	3 (19)	0	
d	0	2 (10)	
e	0	2 (10)	
f	3 (19)	3 (14)	
a + e	1 (6)	1 (5)	
a + c	1 (6)	1 (5)	
a + b + d	1 (6)	1 (5)	
a + b + c	1 (6)	0	
Intraoperative blood loss (ml), mean ± SD	124 ± 61	133 ± 58	0.650
Operation time (min), mean ± SD	103 ± 22	106 ± 25	0.634

*SD* standard deviation

^a^Combined injuries: a: thoracic injuries with multiple rib fractures; b: craniocerebral injuries; c: abdominal injuries; d: ipsilateral clavicle fracture; e: fractures in other sites; f: no combined injuries

### Primary outcome

At 3 months after surgery, compared with the straight reconstruction locking plate group, the Y-type locking plate group showed higher CSF scores (82.9 ± 3.5 vs. 79.3 ± 4.4, *P* = 0.01) and lower DASH scores (9.5 ± 2.5 vs. 12.7 ± 3.9, *P* = 0.008) (Table 2). There were no differences at 6 and 12 months.

**Table 2:** CSF and DASH scores in the 37 patients with scapular fracture at 3, 6, and 12 months after surgery

Variables, mean ± SD	Y-type locking plate *n* = 16	Straight reconstruction locking plate *n* = 21	*P*
3 months after surgery			
CSF	82.9 ± 3.5	79.3 ± 4.4	0.012
DASH	9.5 ± 2.5	12.7 ± 3.9	0.008
6 months after surgery			
CSF	92.4 ± 4.2	90.5 ± 4.3	0.187
DASH	5.5 ± 2.7	5.8 ± 2.6	0.716
12 months after surgery			
CSF	95.8 ± 2.5	94.1 ± 3.3	0.105
DASH	4.2 ± 2.3	4.8 ± 2.5	0.391

*SD* standard deviation, *CSF* Constant Shoulder Function, *DASH* Disabilities of the arm, shoulder, and hand

### Secondary outcomes

All surgeries were performed successfully. There were no differences between the two groups regarding intraoperative blood loss (*P* = 0.65) and operation time (*P* = 0.634) (Table 1). The incisions displayed grade A healing in all patients. There was no case of delayed healing, infection, delayed union, or nonunion. As a result of muscle injury during incision, the infraspinatus muscle and teres minor were partially atrophic in some patients (Y-type locking plate group: *n* = 8; straight reconstruction locking plate group: *n* = 11).

There were no complications such as plate rupture and screw prolapse during the 1-yearfollow-up. At 12 months, there were two patients with mild shoulder pain in the Y-type locking plate group and six in the straight reconstruction locking plate group. No patients asked for plate removal, probably because of the short follow-up. The patients reported no influence on daily life and work. Muscle strength was normal.

Regarding the patients with pain in the Y-type plate group, the first patient was a 30-year-old male with fractures of the scapular body and glenoid neck, accompanied with coracoid fracture, caused by a car accident. The patient was in bed for 15 days before operation. A Y-type locking plate was used for fixation of the scapula and triangular bandage suspension was applied for 4 weeks for conservative treatment of the coracoid fracture. The second patient was a 57-year-old female, with fractures of the scapular body and glenoid neck accompanied by fracture of the peak, caused by a falling accident. The patient was in bed for 6 days before operation. A Y-type locking was used for fixation of the scapula and a triangular bandage suspension was applied for 4 weeks. Both patients were with injuries of the superior shoulder suspensory complex. The slight pain in these two patients could be associated with movement restriction and the injuries of the superior shoulder suspensory complex.

Regarding the patients with pain in the straight reconstruction plate group, all were males. The mean age of the patients was 50.5 ± 6.3 years and the time in bed before operation was 11.5 ± 4.0 days. Two patients also had clavicle fracture, one had scapular peak fracture, and the other three had comminuted fractures of the scapular body and glenoid neck. All six patients were with injuries of the superior shoulder suspensory complex, which damaged the stability of the shoulder joint. In addition, shoulder joint movement restriction after operation could have contributed in inducing shoulder joint dysfunction and pain.

## Discussion

The current treatment of severe displaced scapular fractures with locking plates is challenging because of the shape of the scapula and the patient outcomes are suboptimal, especially for those with extra-articular fractures [10]. There is no study about the Y-type locking plate for the treatment of scapular fractures. Therefore, this study aimed to compare the functional outcomes of the Y-type locking plate vs. the straight reconstruction locking plate for the treatment of severe displaced scapular fractures. The results showed that open reduction using the distal humeral Y-type locking plate could achieve better short-term functional outcomes (3 months) than the straight reconstruction locking plate for severe displaced scapular body and neck fractures, but there were no differences in outcomes between the two groups at 6 and 12 months.

For severe displaced unstable fractures involving the scapular body and neck, the common internal fixations include 3.5-mm locking plate, 2.7-mm reconstruction locking plate, distal radius T-type plate, calcaneous deformed plate, and microplate [3–5, 7, 13–16]. Previous studies reported that for this type of fracture (AO/OTA 14-A3.1 or 3.2), the use of the medial and lateral border [14] and multiplate fixation [5, 8] can achieve good shoulder functions. Ao et al. [14] compared the use of single lateral and medial-lateral plates for the treatment of scapular fractures, and the single lateral plate treatment achieved good clinical results, shorter operative time, less blood loss, and fewer plate-related complications. Burke et al. [22] found that the sclerotin of the base of the scapula spine and lateral border of the scapulae and scapular neck are relatively thick, and are used for anatomic sites of internal fixation. In the present study, the short-term (3 months) shoulder joint function after Y-type locking plate internal fixation surgery was better than with the straight reconstruction locking plate. This difference disappeared at 6 and 12 months.

The Y-type locking plate is relatively close to the shape of the scapula (vaguely triangular). One of the proximal Y-sides was fixed on the glenoid neck of the scapula; the other side was fixed on the base of the scapular spine, while the distal side was fixed on the lateral border of the scapula, forming a triangular multi-planar fixation and fixing the unstable fracture. In addition, using a 3D printing model, the plates can be pre-bent to the actual shape, improving surgical time. Nevertheless, the short- and mid-term outcomes were similar between the two groups.

The modified Judet approach was selected for all patients, but it is known that the dissection of the posterior part of the deltoid muscle during the Judet approach [15] leads to longer immobilization (6 weeks) and muscle trauma [3]. Since the integrity of the deltoid muscle is necessary for the normal function of the shoulder joint, and since deltoid muscle injury often causes shoulder pain, weakness in outreach, and reduced mobility, avoiding as much trauma as possible to this muscle can affect the functional outcomes after surgery [23–25]. Because of those reasons, we routinely create a sub-muscle tunnel by opening a window using a rubber strip to pull the posterior part of the deltoid muscle and infraspinatus muscle before fixing the plate. In addition, postoperative shoulder exercises were performed as early as possible in order to reduce complications caused by shoulder immobilization. Therefore, in the present study, there were no deltoid muscle atrophy, weakness of shoulder abduction, and significant reduction in shoulder motion range. This could explain, at least in part, the good functional outcomes observed in both groups after surgery.

The scores of the shoulder joint functions were not significantly different between the two groups at 6 and 12 months after operation, suggesting that for fractures of the scapular body and glenoid neck, a Y-type plate allows for early functional exercises. Clinical healing of the fracture was achieved at 3 months after surgery and the scapula was whole in all patients. The supporting effects of the plate were probably gradually replaced by that of the scapula. The functions of the shoulder joint were not significantly different between the two groups during endurance and strength exercises of the shoulder joint, no matter which type of plate was used for fixation.

It should be noted that for the patients who received the Y-type locking plate, the postoperative exercises began earlier and with a higher intensity than in the straight reconstruction locking plate group, based on the assumption that the Y-type locking plate achieved a stronger fixation. Nevertheless, no complications were observed and the 12-months outcomes were similar to that of the straight reconstruction locking plate. The postoperative management could be responsible, at least in part, for the better 3-month outcomes in the Y-type locking plate group, but it will have to be examined more closely.

The strengths of the study are that the study population was relatively homogeneous in term of fracture type and that methods were used to minimize the surgical trauma. On the other hand, the study has limitations. It was a retrospective study, the sample size was limited, the follow-up was short, and there were no comparisons with other fixation methods. In addition, the selection of the internal fixation was based on the surgeons’ experience and preference, fracture type, and the patients’ preference (mainly potential complications), rather than randomization. Furthermore, the postoperative management was different between the two groups, based on the assumption that the Y-type locking plate achieved more solid fixation than the straight reconstruction locking plate. Finally, the outcomes were observed by the surgeons themselves and not by independent assessors. Therefore, the conclusions should be taken with caution in the light of those limitations. Of course, the generalizability of the study is limited by the inclusion of patients with only two types of fracture, i.e., AO/OTA 14-A3.1 or 3.2.

## Conclusions

This study suggests that open reduction using the distal humeral Y-type locking plate can achieve better short-term functional outcomes (3 months) than the straight reconstruction locking plate for severe displaced scapular body and neck fractures, but the outcomes are similar at 6 and 12 months. The use of the Y-type plate could be explored for other types of scapular fractures requiring surgery. In addition, formal clinical trials should be performed.

## Abbreviations

CSF: Constant Shoulder Function
DASH: Disabilities of the arm, shoulder, and hand
